# Design and implementation of a Community Expert Group comprised of people with lived expertise of homelessness at an academic health research center: a program description and analysis of challenges

**DOI:** 10.1186/s40900-025-00733-z

**Published:** 2025-05-15

**Authors:** Ayan A. Yusuf, Victoria Hatfield, Katherine Francombe Pridham, George Da Silva, Rene Adams, Daniela Mergarten, Veronica Snooks, Frank Crichlow, Stephen W. Hwang

**Affiliations:** 1 MAP Centre for Urban Health Solutions, St. Michael’s Hospital, 209 Victoria St, Toronto, ON M5B 1 T8 Canada; 2 Realize Canada, Toronto, ON Canada; 3 South Riverdale Community Health Centre, Toronto, ON Canada; 4 University of Toronto, Toronto, ON Canada

**Keywords:** Community, Community Experts, Homelessness, Research Engagement

## Abstract

The involvement of communities with a stake in healthcare research is often limited, and attempts to increase their participation and to create shared decision-making partnerships are often hindered by structural barriers. In this paper, we describe the design and implementation of a Community Expert Group comprised of people with lived expertise of homelessness at an academic health research center. We detail the group’s model, guiding principles, governance structure, and activities, and discuss institutional challenges encountered over the course of this partnership. We report that the lack of policies and practices in academic research institutions to support long-term collaboration with community experts makes it challenging to define their scope and role, often requiring individual research teams to fill this gap.

## Abstract (plain language summary)

Academic researchers have largely carried out their work in a top-down approach, with community's role often being relegated to being assigned as research participants or through minimal public consultation. For decades, communities with lived experience have called for their perspectives to be incorporated into research on issues affecting them. While there are several widely accepted approaches to address the harmful trajectory of leaving out community voices, less know about the practical implementation and challenges within large academic research institutions. In this paper, we describe the design and implementation of a Community Expert Group comprised of people with lived expertise of homelessness at an academic health research center. We detail the group’s model, guiding principles, governance structure, and activities, and then discuss institutional challenges we encountered over the course of this partnership. We report that the lack of policies and practices in academic research institutions to support long-term collaboration with community experts make it challenging to define their scope and role, often requiring individual research teams to fill this gap.

## Background

Research examining the experiences of people experiencing homelessness (PEH) has been critiqued for taking a top-down approach in which academic researchers fail to consider the contextual factors of homelessness [1]. Such an approach lacks the critical voice of the communities affected by the research. It also has the damaging consequence of framing PEH as poor, disadvantaged, and “other,” further entrenching systemic marginalization of PEH in society [1, 2]. This approach can also position the cause of homelessness at the individual level rather than the structural level, thus negatively influencing public perceptions and policy [3].

Homelessness is a major social and health issue in cities around the world. In Canada, more than 235,000 people experience homelessness each year [4]. In Toronto, Canada’s largest city, there are over 12,000 people who experience homelessness on any given night [5]. Limited stock of affordable housing, low vacancy rates, and structural barriers to accessing health care and social support contribute to a lower quality of life for PEH. As a result of these systemic and structural barriers research has found that PEH have high rates of premature death [6], injury [7], chronic illnesses [8], infectious diseases [9, 10], and mental health and substance use disorders [11].

Studies indicate that PEH experience systemic barriers to accessing primary care, use emergency services more frequently than their housed counterparts, and have longer hospital stays and higher total healthcare expenditures [12–15]. PEH have reported that interactions with the healthcare system are often stigmatizing, shaming and discriminatory [16, 17], to the extent that they sometimes avoid seeking needed healthcare services [18].

In recent decades, there have been important movements within the community of people with lived/living experience of homelessness, housing precarity and poverty to demand increased inclusion in decision-making related to research and clinical care programs that affect them. This development is in large part a result of movements such as “Nothing About Us Without Us” that call for increased involvement roles of people with lived expertise in contextualizing research [19]. Alex Nelson, an activist-researcher with lived experience of homelessness, emphasizes the importance of centering “the profoundly transformational knowledge of oppressed and exploited people” in challenging research and policy that reinforce the marginalization of PEH [20]. Research that engages communities closely impacted by the issue being studied have been shown to be more likely to meet the project’s objectives, provide new perspectives in data interpretation to shape policy change, and improve outcomes of interventions [21–23]. In homelessness research, partnering with communities with lived experience of homelessness and housing precarity is considered instrumental in aligning research and policy goals with community priorities and may produce more human rights based solutions to housing [24, 25].

There are several forms of community-partnered research, such as Community-Based Participatory Research (CBPR) and Participatory Action Research (PAR). These types of research have been employed to address the harmful trajectory of leaving out community voices and make more effort to center self-identified research priorities. For decades, some of these approaches have gained global practice largely as a result of work by the likes of Colombian sociologist Fals Borda and Brazilian educator Paulo Freire, among others, who have challenged traditional academic research practices that reinforce marginalization [26, 27]. In Canada, there are several major initiatives to support these endeavours. The Strategy for Patient-Oriented Research (SPOR) was created by the Canadian Institute of Health Research to encourage partnership between researchers and patients at all stages of research and care to ensure that the patients set key priorities [28]. The core areas of engagement in this model include patient engagement in governance and decision-making, capacity building for patient engagement and the creation of tools and resources for patients and researchers. The Spectrum of Public Participation, created by the International Association for Public Participation, is another model used by institutions to define levels of community engagement. The spectrum in this model ranges from simply informing patients about issues affecting them to empowering them to have decision-making authority and ownership in the partnership agreements [29]. Despite the numerous models outlining best practices for community-partnered research, there is a dearth of literature detailing the implementation of these approaches in the context of homelessness and research performed at academic healthcare institutions.

The objective of this paper is to describe the design and implementation of a Community Expert Group (CEG) comprised of people with lived expertise of homelessness that guides and advises a program of research on homelessness, housing and health within an academic healthcare institution. It is a reflection of our learnings and is co-authored by several members of CEG, some of whom have contributed to co-designing the CEG model. We first detail the group’s model, guiding principles, governance structure, and activities, and then discuss challenges encountered over the course of this partnership.

## Methods

### Overview

This work occurred in the context of a health equity research center based within an academic hospital setting in Toronto, Canada. Our team’s research program over the past two decades has focused on designing, implementing, evaluating and advancing interventions to improve the healthcare delivery and health outcomes of PEH and facilitate their transition into housing. This includes individuals living on the street, in encampments, or in shelters. In 2020, the principal investigator leading the research team initiated the development of a Community Expert Group (CEG) with the aim of ensuring that people with lived expertise of homelessness were included in the research program on an ongoing rather than project-specific basis. This approach was taken to avoid simply going through the motions of community consultation in research but rather to create a space to encourage academic researchers to seek relevant perspectives, highlight broad community priorities, facilitate community connection, and build opportunities to strengthen community research knowledge and skills for both academic researchers and community experts. Prior to initiating the CEG, the research team had informal collaborations with community organizations and advocates within the homelessness and housing sector, and on a few occasions, established a time-limited advisory group for the duration of a specific research project. Advice from community partners and reflections from our research projects led our team to undertake a new effort to include communities that are affected by homelessness research who are excluded from academic institutions. Our goal was to form a well defined mutually beneficial partnership that aligned with community interest.

The CEG was initially formed in 2020 with four members and was eventually expanded to eight members. CEG members are people with lived and living expertise of homelessness who had knowledge and experience navigating health care and social services in Toronto. The group had members with diverse social locations and included individuals who identified as Black, Indigenous, and/or racialized and members of the 2SLGBTQIA + community and with most having longstanding experience influencing homelessness and housing policies in Toronto.

One research staff member dedicates a substantial portion of their time to supporting the CEG's work by managing communications within the group, coordinating and attending meetings of the CEG, facilitating training and co-learning opportunities, and connecting the CEG to researchers within the research center. In addition, person with lived experience of homelessness was engaged as a facilitator to support the group’s work, help build relationships between research staff and CEG members, assist with establishing a more balanced power dynamic and support members in negotiating their roles and developing effective collaborations.

### Recruitment

Members of the CEG were recruited through collaboration with a broad range of community organizations working in the homelessness service sector in Toronto, most of which are members of a network working to achieve and maintain zero homelessness in Toronto known as the Toronto Alliance to End Homelessness. The research team contacted organizations through email and attended community meetings to share information about the initiation of the CEG. A flyer provided information about the group’s goals, expected responsibilities, and potential benefits of participation such as research training. A low-barrier application process was created. Applicants were asked to respond via email or phone and answer questions about their experiences with homelessness and housing advocacy and about their interests in research. There was no request for a traditional resume. Research staff met with all interested applicants, either in-person or virtually, using an informal format. The conversation during the meeting covered applicants’ responses to application questions. We also discussed applicant’s availability to participate in CEG activities, the resources they would need to facilitate their participation, and their preferred method of compensation for their involvement. Our current institutional compensation options include gift cards, cash, e-transfer, cheques or electronic fund transfers. We discussed each option with applicants as well as any associated constraints such as potential delays in receiving payments.

Since 2022, additional CEG members have periodically been recruited to increase diversity of representation, interests, and perspectives. Recruitment of new members has helped to foster new relationships with communities affected by homelessness and to expand the group’s areas of expertise and skill sets [30, 31]. Group expansion has also supported sustainability, a key principle of community-partnered research implementation [32, 33], as the ability of individual CEG members to engage in the work of the group has varied substantially over time due to health issues, life circumstances, and competing commitments. Nevertheless, we acknowledge no one group of people can represent an entire community with many intersecting identities. This has been highlighted in our annual reflection, in which CEG members are asked to share what perspectives they would like to see included in the group. Responses have included younger people with homeless experience and people with more recent homeless experience. Over the years, the group members have participated in the expansion of the group by sharing recruitment efforts with their social and professional networks.

### Governance structure

Establishing a governance structure for the CEG and creating an equity-informed shared mandate began with the consideration of two key factors. First, we sought to actively recognize existing structural inequities (i.e., institutional racism and discrimination) that might make individuals hesitant to collaborate with a research team situated within an academic hospital setting. Second, we acknowledged the inherent power imbalances in academic-community partnerships.

To address these barriers, in the first month of our partnership, the group co-wrote terms of reference. The terms of reference outlined the group's purpose and allowed all parties to collaboratively define the scope of the partnership, roles and responsibilities, a code of conduct, and a system of compensation early in the partnership. Traditional committee roles were forgone to avoid creating hierarchies in the group and prevent members from burning out in a particular role. Rather, a more flexible structure was adopted in which responsibilities of each member change depending on their involvement in different research projects [34].

While initiating partnerships through an equity-informed framework is necessary, shifting traditional power dynamics between researchers and community members does not occur overnight and requires a degree of flexibility and critical reflexivity. Since the establishment of the group, members have participated in multiple one-on-one and group meetings to refine the terms of their work and have made amendments as needed. This co-creation process was necessary due to the aforementioned historical distrust of institutional partnerships among the community, specifically, experiences of tokenism and discrimination that have either minimized or excluded community voices [35, 36].

## Results

Over the past four-years following its inception, the CEG worked to meet three objectives: (1) to engage in research training to increase the group’s capacity to engage in research and with researchers; (2) to advise academic researchers at various stages of their projects related to homelessness on how to better work with the community; and (3) to identify and work on priority areas for new research projects on housing, homelessness, and health. Key aspects of the design and implementation of the CEG are reported below, with a focus on operational considerations, activities, and outcomes.

### Operational considerations

#### Meeting and work activities design

The CEG typically meets twice a month. Initial meetings of each year are dedicated to refining the terms of reference and co-developing an action plan to meet the three objectives of the group. Each meeting begins with a land acknowledgment, followed by a reading of a co-developed group agreement, a brief check-in with everyone, and review of the agenda for the meeting that has been prepared by research staff and the facilitator. Typically, the meetings are two hours long and require some advance preparation work by CEG members. Outside of these working meetings, CEG members meet for informal discussions with the dedicated primary research staff member and the CEG facilitator, providing opportunities to bring forth new ideas for group functioning and activities. Additionally, these informal meetings provide opportunities for social networking among group members.

To create a more inclusive and supportive environment that would encourage members to express themselves freely, it was initially planned to host CEG meetings at a shared work space within a community agency. The space was considered a more neutral environment for community collaboration than the academic health center’s research office; however, the shared working space became unavailable due to COVID-19 restrictions. Most CEG meetings in 2021 and 2022 were held virtually, and members without devices to support video conferencing software were provided with either a tablet or a laptop. As COVID-19 restrictions were lifted, the group was asked about preferred meeting locations, and reflecting the relationships with the research team that had been established by that point, all members were open to meeting at the team’s research office for in-person meetings. Currently meetings are hybrid to allow some members to participate virtually when there are challenges attending in-person.

#### Compensation and support

Members of the CEG were compensated for attending meetings at a rate of $30 (Canadian) per hour starting in 2020, in alignment with other research teams across the research institution. In 2023, conversations with the group revealed the importance of revisiting compensation rates to adjust to raising yearly cost of living, and the amount was therefore increased to $35 per hour (Canadian) for 2024. CEG members are also compensated for meeting preparation time, project-specific meeting time outside of scheduled group meetings, and the cost of public transportation to and from in-person meetings. Over the years, we have revisited the preferred method of compensation and payment schedule with all members and made adjustments to meet individual needs. For instance, one member requested to be paid every few months so they can receive a lump sum. At in-person meetings, refreshments are provided. During the initiation of the group and subsequent one-on-one check-ins with each member, we discussed what material support CEG members might need to participate in meetings and carry out their work. As result, we provided some members with a tablet or laptop to use for the duration of their membership in the CEG. In an effort to maintain a low-barrier approach, devices were delivered to members’ preferred location with email confirmation of delivery. Considering the ongoing living experience related to housing and health, over the years, the research team has also extended assistance to help members navigate social and health services when needed.

### Activities and outcomes

Meetings are broadly classified into three categories: research feedback sessions, capacity-building sessions, and priority setting and new project development sessions.

#### Research feedback sessions

Research feedback session are an opportunity for researchers to seek guidance and feedback from the CEG at various stages of their work related to homelessness (e.g., developing new research proposals, operationalizing data collection, data analysis, or planning for knowledge translation). A recent example of this is the CEG’s feedback on the dissemination of results from a project evaluating a supportive housing program, and they have also been acknowledged in several publications from the research team on other projects [37–40].

Researchers who engage the CEG for feedback sessions are typically affiliated with the academic hospital where the CEG is based, but some researchers are affiliated with other institutions in Toronto. To begin this process, researchers submit a written request for feedback to the primary CEG research staff member and facilitator for review, who then shares the request with the group. In some instances, the primary research staff or the facilitator may meet with the researcher to provide guidance on completing the written request for feedback or review expectations for the feedback session. The researcher provides an overview of the research project, identifies community priority the team is taking into consideration, and the specific advice or feedback they are seeking from the CEG. This process also provides an opportunity for the researcher to reflect on their research approach and the relevance of their project to the priorities and needs of the community. Additional materials such as interview guides, research proposals, and community consultation plans are usually shared with CEG members to review before the research feedback session. Within a week of submitting materials, a 60- to 90-min research feedback session is held with the CEG. Members participate in different ways; some provide detailed written feedback before the meeting, while others provide verbal feedback during the meeting. Bidirectional communication between research staff and CEG members both before, during and after the meeting is essential to the success of research feedback sessions, as it generates richer conversations and challenges power imbalances. Moreover, bidirectional communication is also a key ingredient to fostering respect and mutual trust [41–43]. This type of bidirectional communication takes shape in academic researchers respectfully questioning feedback they receive from community experts, providing additional context when receiving feedback, providing updates on how feedback is being implemented and explaining institutional or project limitations. When this does not occur, the process can feel inauthentic and will leave community experts wondering whether solutions provided will be used or discarded. Occasionally, the CEG facilitator posed follow-up questions to academic researchers to ensure the discussion remains a dialogue.

In addition to providing feedback on research projects, CEG members provide feedback on the process itself, resulting in changes that make the process meaningful for everyone. These sessions also result in rapid response for members looking for new opportunities to be involved in research projects on an ongoing basis as academic researchers will often present opportunities for participation at the end of the session. This includes opportunities to be involved in research as researchers with lived experience, contribute to manuscript writing, and participate on advisory boards. One member shared that a referral to another research team working on an issue that affected them opened up opportunities to gain new skills and utilize existing skills, be more involved in community advocacy, participate on panel discussions and conferences, and expand their research network.

#### Capacity building

Capacity building is foundational to ensuring that community partnerships in research are mutually beneficial. Moreover, it is an important way of sustaining community partnerships and increasing knowledge exchange between research staff and community partners. Israel and colleagues [32] outline co-learning and capacity building as the “reciprocal transfer of knowledge, skills, and capacity” [44]. Academic researchers can learn from community members by increasing their understanding of the community’s strengths and the issues they face, while community members can learn from academic researchers by building their formal research skills. Early in the group’s formation, we asked the CEG members what research knowledge they would like to gain while working with the research team, which helped in the preparation of training materials. Additionally, members complete an end-of-year reflection survey every year to identify areas for future training. To date, CEG members have participated in over 10 research-training sessions to expand their knowledge of research processes and capacity to conduct research activities. These training sessions were mainly designed and delivered by the research team and covered topics on research design and methodologies, ethics, qualitative interviewing, and how to conduct literature searches. The group also participated in sessions designed to improve group function, focusing on topics such as anti-racism and mindfulness trainings. Most of this training occurred in the first two years of CEG, which resulted in more frequent meetings and opportunities for rapport and relationship building between the research team and CEG. As previously reported, positive impacts from capacity-building not only include improved formal research capacity among community members and research partners, but also increased trust, meaningful engagement and collaboration, and enabled access to information to challenge traditional power structures [45, 46]. In annual reflections, CEG members have shared that training also strengthen their ability to advocate for their peers who participate in research.

#### Priority setting for new project and knowledge sharing

Priority setting meetings are designed to help align community priorities with research plans. Research is often driven by researchers’ interests rather than community priorities, therefore, these sessions are opportunities for the CEG group to discuss community issues that are important to them and strategize ways in which the group and research teams can have a more meaningful impact on the lives of people experiencing homelessness. For instance, early in the group's formation, members identified that legislation on rooming house in Toronto was their community priority, which led the research team to prioritize advocacy to reform rooming house legislation. Priority setting meetings are also an opportunity to discuss ideas for dissemination and increasing knowledge to action efforts. Traditionally, research dissemination takes place through academic journals or conferences. This process primarily benefits researchers, reinforces their ownership of the knowledge production process, and fails to reach service providers and policy makers who can translate findings into practice [47, 48]. Community-partnered research principles emphasize the need for knowledge translation that is accessible, benefits everyone involved, and leads to transformative social change [32, 49]. Over the past four years, CEG members have encouraged knowledge to action efforts through participation in webinars, open letters, policy briefs, and the co-development of guidelines for community partnered research.

## Discussion

There are numerous approaches to working with community experts in research. However, implementing equitable partnerships with communities affected by health and social research is challenging, especially in institutions such as hospitals [50, 51]. Over the past four years of implementing the CEG, we have encountered several challenges, some of which are highlighted below. These challenges have resulted in important learnings and opportunities to further challenge persistent barriers that impede equitable research partnerships..

### Institutional hierarchy and the scope of work

Governance structures for groups such as the CEG are of great importance, given the history of traditional health research approaches and the unwillingness of some researchers to relinquish power [52]. Community experts often enter the research process with pre-existing lived knowledge around hierarchies of authority, which can impact their relationships with academic researchers. This can deter community experts from engaging with researchers, as they may feel they have limited influence over the research process [35], may not benefit from it, or may be actively harmed by it [53].

Moreover, it is widely agreed that creating an equitable power dynamic is foundational to ensuring that the relationship between researchers and the community is collaborative and allows everyone involved to benefit from the partnership mutually [32, 54, 55]. It also requires long-term commitments to meaningful engagement and funding, which are necessary to build trust and cultivate relationships in which all group members feel comfortable to voice ideas, questions and concerns [32, 56, 57].

Large institutions like hospitals have hierarchical organizational structure that dictates employment status and corresponding entitlement to resources and benefits. In this hierarchical approach, community experts who are often limited to being brought in as external consultants due to limitation in traditional hiring practices (e.g., electronic resumes and criminal record checks requirements) or funding limitation. This means that in some cases community experts are the last to be considered in assembling a research team with roles that lack clarity [58].

The long-term commitment of resources to sustain the CEG over time contributes to overcoming such institutional challenges and has helped strengthen our relationship with the group. Much of the primary research staff’s time is dedicated to ensuring that work with the group is meaningfully engaging. Over the years, this has included initiating projects that the group has identified as a priority with the support of other staff and students. Additionally, using our terms of reference, we have committed to paying special attention to defining the roles and responsibilities of CEG members to mitigate power differentials between our research staff and community experts.

Despite these efforts there are challenges in onboarding and integrating the group within the broader institution. This is due to a lack of the necessary organizational policies needed to sustainably support community-partnered research, namely in employment opportunity, equitable compensation, and resources provision beyond what is required for their work. The lack of clear and standardized mandates in these areas leads to teams developing their own solutions to arising challenges, and can result in a lack of accountability when things do not go as intended.

### Recruitment practices

Our community experts are engaged as independent consultants, and our research funding allows us to compensate them for their time and support their technology and public transportation to and from CEG-related meetings and events. The funding also provides dedicated staff time and a facilitator with lived expertise to help support effective work. Hiring practices in academic research settings have barriers such as educational requirements and criminal background checks, which limit employment options for individuals who want to contribute to research that directly affects them. Moreover, traditional working arrangements and specific skill requirements, such as computer literacy or institutional requirements, can exclude some community experts from positions with task they can otherwise perform. The lack of institutional hiring practices that are inclusive of community experts from diverse backgrounds can also foster the performative inclusion of systemically marginalized people and lead to tokenism [58]. Additionally, it normalizes the perception that communities affected by research are at the bottom of the hierarchy and devalues their contribution. To uphold our efforts to counter these challenges and encourage reciprocity, we regularly share external opportunities with the CEG to further develop research skills and expand their network. Additionally, CEG members are prioritized for opportunities to be involved in specific projects within our research program in different capacities, such as research assistants or advisory members.

Another issue in recruitment practices for groups like the CEG that are situated within academic institutions is ensuring that that opportunities for engagement are well-defined and align with community experts’ interest and expertise [50]. People with lived expertise often experience tokenism in academic research settings and are less likely to apply to open opportunities when they are not familiar with the research team. This makes it difficult to build new relationships and likely hindered our past recruitment efforts to reach more individuals with differences in culture, values, and perspectives as they did not garner many applicants.

An equitable partnership between researchers and non-academic community partners begins with asking the important question of who represents “the community” most affected by the research project [59–61], and then committing to genuine relationship-building, which takes a lot time and resources. Populations of people that are “othered”, including PEH, are often homogenized as one monolithic community by academics. In our work, we actively seek diversity of perspectives in issues affecting people experiencing homelessness and housing precarity as there are a myriad of intersecting issues they face, resulting in different concerns and research interests [31, 59, 62]. Dedicated institutional funding and resources to building community relationship would facilitate overcoming these challenges especially for research teams with limited research funding streams.

### Compensation

Compensating community experts in research partnerships is largely agreed upon to be a good practice in research and is strongly encouraged [59]. However, the application of equitable compensation practices that meet the needs of individuals varies from research team to research team and, in most cases, needs more institutional direction [63]. These institutional compensation barriers show up in how payments are processed for community experts, particularly in determining pay rates and methods, and implications for people receiving other sources of income-namely government assistance such as Ontario Works.

Compensation needs should be aligned with a commitment to timely payment for completed services, as outlined in many community-partnered research frameworks. For CEG members, compensation often provides supplemental income, and in the past some members have experienced delay in payment due to institutional finance policies. Similarly, restrictive government assistance programs, which are often criticized for low benefits payments, can scrutinize any additional income received, and therefore working as a CEG member can risk entitlement to those benefits that have income-based requirements [64]. Community researcher with lived expertise who receive government assistance are often worried about risking their eligibility for housing and health benefits, which can deter them from participating in research or avoid claiming their payment [64]. Ongoing discussions with the CEG regarding compensation options and policies and participating in advocacy to challenge institutional shortcomings have helped us address these concerns.

### Social and mental health support

There are insufficient avenues to respond to the lived and living experience of members in the group, such as housing loss, mental health support, and health and social service navigation. Over the years, our team has struggled with defining the scope of support beyond assisting the group in carrying out their duties effectively, especially when it is not well defined at an institutional level. This is challenging because, in their roles, the group uses their lived experiences to guide our research direction and often recount difficult and emotionally triggering experiences. While we engage in ongoing individual check-ins with members going through difficult times and offer informal support to navigate social and health services, there are instances when we cannot meet individual needs in certain areas, such as housing and mental health. Therefore, at CEG meeting sessions, we encourage everyone to protect their emotional wellness, and there is no expectation to disclose difficult experiences to carry out their duties. Moreover, during difficult discussions we have learned to not regulate how people choose to express themselves. Teams across our institution and other organizations do not always have the time, knowledge, and resources to support community researchers with lived expertise adequately, which further highlights the need for organizations to have a comprehensive strategy and well-defined scope of support and benefits for community experts engaged in research. As such, shifting hiring and benefit provision practices for community experts is essential at an institutional level.

### Managing group dynamics

This collaboration brings together individuals from diverse social locations and with lived and living experiences of systemic marginalization. Within this setting, interpersonal relationships between the CEG group members and power dynamics between researchers and group members have required us to reimagine ways to cultivate accountable space that allows people to express their evolving insights, while also making efforts to protect people from conversations that may re-traumatize them. In past yearly reflections, some members of the group shared that they believed the CEG’s co-developed terms of reference, group agreement, and code of conduct have helped to foster a shared accountable space that encourages members to share their insights, while also being aware of differing perspectives. Additionally, the group has participated in training activities and debriefs to help support group function and dynamics. The research team is also responsible for upholding the roles and responsibilities outlined in the terms of reference to minimize harmful power dynamics. This approach has helped build trust and a strong CEG membership over time.

Nevertheless, developing strong group dynamics has not been linear, occasionally resulting in challenging interpersonal interactions. Most of the members have worked together on other advocacy projects and, therefore, already had established relationships, which sometimes contributed to conflict due past unresolved issues. One of the challenges that has arisen is triggering conversations. From time to time, the group discusses challenges that PEH face, which can include reliving difficult past events. Instances like this can emotionally trigger members. Over the years, we have struggled to strike a balance between creating a space that allows people to share experiences while also providing a psychologically safe environment for everyone involved. In instances when members were unable to maintain the agreed group terms, they were respectfully asked to take some time away from the group to address any underlying challenges. We also continually revisited the group’s code of conduct and had one-on-one check-ins to address any concerns. We also found informal meetings dedicated to socializing helped encourage social connection and improve group dynamics.

## Conclusion

Over the past four years, CEG members have contributed to research projects, influenced processes of working with people with lived expertise at the research centre, advocated for systemic change in homelessness, housing, and health and contributed to writing of this paper. Our experience shows that sustainably working with community experts in research takes a lot of time and resources, as previously reported in other research [65]. Our approach to working with CEG was not linear, however, our learnings add to other research by others including Malenfant et al. [24] and Padwa et al. [66], who have highlighted the importance of working with people with lived experience of homelessness in creating housing and health solutions that respond to peoples lived realities. We found that operationalizing CEG objectives within an academic research center has several challenges. Research institutions lag behind in hiring practices and the availability of support for people with lived expertise engaged in research. As a research team, this made it difficult to define the scope of our work with the CEG. Over the years, we have also encountered challenges of working as a group in a setting that is prone to power imbalance and where many people with lived experience have experienced racism and discrimination. We have learned that continually revisiting our terms of reference, having one-on-one check-ins, engaging in bi-directional communication, and creating meaningful work that interests the group have enhanced our collaboration. Our experience demonstrated that engaging community experts in research at academic institution is an iterative process and requires critical reflections and flexibility to change. Other academic researchers who are considering this work should adopt a process that includes people with lived experience in designing every aspect of their partnership and co-create solutions that challenge existing institutional barriers.

## Data Availability

No datasets were generated or analysed during the current study.

## Abbreviations

CEG: Community Expert Group
PEH: People Experiencing Homelessness
